# Functional Profile of Older Adults Hospitalized in Rehabilitation Units of the National Network of Integrated Continuous Care of Portugal: A Longitudinal Study

**DOI:** 10.3390/jpm12111937

**Published:** 2022-11-21

**Authors:** César Fonseca, Ana Ramos, Lara Guedes Pinho, Bruno Morgado, Henrique Oliveira, Manuel Lopes

**Affiliations:** 1 Nursing Department, University of Évora, 7000-801 Évora, Portugal; 2 Comprehensive Health Research Centre (CHRC), University of Évora, 7000-801 Évora, Portugal; 3 Hospital Center of Medium Tejo, 2304-909 Tomar, Portugal; 4 Hospital Garcia de Orta, 2805-267 Almada, Portugal; 5 Institute of Telecommunications, 1049-001 Lisbon, Portugal; 6 Polytechnic Institute of Beja, 7800-295 Beja, Portugal

**Keywords:** aged, functional status, quality of health care, hospitalization, self-care, rehabilitation

## Abstract

Background: The success of healthy aging depends on the ability of countries to study and act on frailty in the elderly, control chronic diseases, improve functional capacity and prevent cognitive decline and social interaction. Aim: (1) to evaluate the functional profile of older adults hospitalized in a Unit of the National Network of Integrated Continuous Care of Portugal; and (2) to assess the relationship between functional profile and age, gender, level of education and emotional state. Methods: Longitudinal study with a population of 59,013 older adults (65 years or older) hospitalized in Medium-Term Care and Rehabilitation Units in Portugal. Results: Older age (≥85 years), no school attendance, low body mass index and presence of sad or depressed mood were predictive factors for a deficit in functional capacity. We identified significant improvements in rehabilitation, but after 210 days of hospitalization, older people incurred a loss of functionality. Total compensation needs were typified by severe deficits in self-care and functional capacity: 47.1%. A moderate deficit was present in 43.1%, and a slight self-care and functional deficit occurred in 9.8% of the individuals. Conclusions: Knowing the determinants of functional capacity and self-care needs will make it possible to define priority intervention groups and implement quality and financing models based on gains in functionality.

## 1. Introduction

With the global trend towards aging and the increase in the number of chronic diseases, there is a concern with the promotion of self-care and self-management of disease as central issues in public health, which can impede economic development and affect the health status of populations [1]. The assessment of the type and degree of dependence on self-care allows the assessment of the health status of people aged 65 or over, which is crucial for planning the care that will be needed [2,3].

The health and functional condition of people aged 65 or over cannot be intuited only by the presence or absence of disease but by the appreciation of all the circumstances that interfere with their well-being and functioning. Broader assessments are responsible for better predictors of survival and quality of life [4]. At the same time, self-care can be understood as an ability for individuals, families, and communities to promote and maintain health, prevent disease and deal with dependence and disability with or without the support of health professionals [5].

The functioning of an individual inevitably involves the complex relationship between the health condition and the contextual factors in which he/she lives. If a change occurs in some of the variables, it may result in a decrease in the individual’s functioning, leading to the need for help from other people to meet the activities of daily living [6]. Activities of daily living (ADL) and instrumental activities of daily living (IADL) are essential for maintaining self-care and an independent and functional life [7].

Dependence, as the inability to perform one or more activities of daily living without additional or complete help from another individual, may be temporary or permanent, meaning that it can be prevented, reduced or resolved in an appropriate context and with appropriate assistance [7,8].

In this sense, in a context where a situation of dependence is installed, there is a need to rehabilitate the individual, promoting their functioning to meet their ADL and IADL, thus restoring their partial or total independence and self-care [6].

Functional rehabilitation, if identified and intervened early, may become only a temporary loss of functioning. This temporary loss of functioning, if rehabilitated, may generate a set of gains in terms of self-care so that sometimes the individual can return to his/her previous level of independence. However, a study that sought to obtain a better understanding of the self-care of older adults who were temporarily rehabilitated in an institutionalized care unit after a lower limb fracture concluded that, if the components “knowledge”, “skills” and “competence” were not observed in the rehabilitation process, they would not develop their self-care [9].

In Portugal, the integration of continued care into the National Health System has become one of the most important reforms, which allows for an extension of care after hospital discharge, allowing the individual to have access to care appropriate to their level of dependence. The National Network for Integrated Continued Care (NNICC) intends to respond to a set of international assumptions defined for long-term care. These assumptions consist of reducing the need for hospitalization and visits to emergency services due to the lack of continued follow-up. The creation of the Network contributes to a reduction in the number of late hospital discharges and greater efficiency of acute care responses, with the provision of continued support services to people in situations of fragility or with chronic illness. The Medium-Term care and Rehabilitation Units (MTRU), part of the National Network of Integrated Continued Care (NNICC), are designed for temporary situations, with the objective of promoting rehabilitation, autonomy and control of the disease process or acute or chronic disability. It is aimed at situations where there is an expected need for hospitalization for between 30 and 90 days. Several services are provided, such as: (a) daily medical care; (b) permanent nursing care; (c) physiotherapy and occupational therapy care; (d) prescription and administration of drugs; (e) psychosocial support; (f) hygiene, comfort and food; (g) socializing and leisure [10].

As the main objective of these units is to rehabilitate people with temporary disabilities, it is essential to assess the evolution of functioning to understand the health gains obtained.

Thus, the aim of this study was: (1) to evaluate the functional profile of older adults hospitalized in a Unit of the National Network of Integrated Continuous Care of Portugal; and (2) to assess the relationship between functional profile and age, gender, level of education and emotional state.

## 2. Materials and Methods

### 2.1. Study Type and Sample

This was a longitudinal retrospective study, with a population of 59,013 older adults (65 years or older) hospitalized in Medium-Term care and Rehabilitation Units of NNICC in Portugal.

### 2.2. Instruments

The Network resorted to the use of an Integrated Assessment instrument from its creation (2006) to 2017, which aims to identify physical, functional, mental, social disorders and life habits, whose results were crucial for the definition of an individual intervention plan, with an emphasis on maintenance and recovery of capacities. The variables constituting the Integrated Assessment Instrument were analyzed: sex, age, health complaints, nutritional status, falls, locomotion, physical autonomy [11], instrumental autonomy [12], emotional complaints, cognitive status, based on the Mini-Mental State [13], social status and habits [14]. The constituent variables analyzed in big data were according to an elaborated concept map that defines self-care, suggested by the literature [3] and statistically validated in this study.

To assess the functional profile, records were used which contained the following components: Walking/Mobility (walking on the street, on stairs, at home/inside buildings), Activities of Daily Living (ADL) (using the toilet, lying down/getting up, dressing/undressing, washing/bathing, controlling the sphincter, feeding); Instrumental Activities of Daily Living (IADL) (preparing meals, washing clothes, homework, shopping, using transport, managing money, taking medication and using the phone) and Cognitive State (time and space orientation). Each item is rated on a Likert-type scale, with the following scores: no problem = 1; moderate problem = 2; severe problem = 3; complete problem = 4. The validity and fidelity of the items constituting the self-care and functional capacity were submitted to Analysis of the Main Components: Mobility (KMO = 0.743; *p* < 0.000); ADL (KMO = 0.885; *p* < 0.000); IADL (KMO = 0.917; *p* < 0.000) and Cognitive State (KMO = 0.593; *p* < 0.000) and Cronbach’s Alpha of α = 0.951 [6]. The results show excellent internal consistency. High correlation of the items constituting IADL and lowest, but acceptable in the cognitive state, probably related to the smallest number of constituent items

### 2.3. Data Collection

Data were collected from the database of the NNICC Medium-Term care and Rehabilitation Units for the period between 1 January 2008 and 27 February 2017. These records were made by health professionals from these units at a national level. Assessment data were collected every 30 days to assess the evolution of the functional profile. All Units of this typology in Portugal were included.

### 2.4. Statistical Analysis

Based on a previous systematic review [3], four components of self-care were identified (mobility: walking; basic activities of daily living; instrumental activities and cognitive status: orientation in time and space), and its suitability was analyzed through principal components and Chronbach’s alpha.

Four indices were also constructed through the statistical weighting of their indicators, that is, by their factor weights instead of the classic arithmetic mean, hence their designation of standardized values [15]. In order to analyze whether the mobility, basic activities of daily living, instrumental activities and cognitive status indices differed in two or more populations, a simple parametric analysis of variance (ANOVA for short Analysis of Variance) was performed. In order to carry out the One-way Anova, the assumption of normality of the distributions was verified; however, it is important to mention that, as this is a large number of cases (N > 30), the breach of this principle does not have serious consequences, invoking for this the effect of the central limit theorem [16]. This analysis was performed on age classes, schooling, body mass index and emotional state, given that they are quantitative variables (Likert-type scale) in K samples (3 or more). The *t*-Student test also serves to test whether or not the means of two populations are significantly different, also requiring that the dependent variables have a normal distribution with homogeneous variances. This test was used to compare the mean of a quantitative variable (synthetic index) with two interdependent samples (sex) [17].

For a better understanding of the evolution of these synthetic indices, which reflect the functional and self-care capacity of the elderly person, over the days of hospitalization, a longitudinal analysis was performed based again on parametric tests (One-way Anova).

Subsequently, the articulation between the analysis of the principal components and the analysis of clusters was carried out, which is an exploratory technique of multivariate analysis, which allows grouping subjects or variables into homogeneous groups regarding one or more common characteristics [17]. Cluster analysis using the hierarchical method was performed to relate the participants’ functional profile with age, sex, education level and emotional state. Due to the large amount of data to be processed in SPSS software, a subsample with approximately 20% of the dataset was considered to obtain the agglomerative coefficients using Ward’s method. Then, to obtain a solution for three clusters, the highest 30 agglomerative coefficients were projected in a graph to visualize their distances and realize the desired solution’s feasibility.

### 2.5. Ethical Procedures

This study was conducted in accordance with the guidelines of the Declaration of Helsinki and was approved by the Ethics Committee of Scientific Research in the Areas of Human Health and Welfare of the University of Évora (report number, 17,036; date of approval, 26 April 2017).

## 3. Results

### 3.1. Socio-Demographic and Clinical Characteristics 

The mean age of the study participants was 79.74 years, and the most frequent were 80 and 82 years. The dispersion from the mean (standard deviation) was 7.205 years. Recoding all ages into age groups, 48.1% of the participants were aged 75 to 84 years, 27.3% were 85 or older, and finally, the smallest proportion (24.6%) were aged 65 to 74 years. The majority were female (58.0%), with 42.0% being male. Table 1 lists the socio-demographic data of the population.

Regarding marital status, there was a great predominance of married people (46.6%) and widowers (36.9%).

As for the professional level of the population, most individuals do not have any type of qualification (70.3%), followed by qualified individuals (23.4%).

### 3.2. Evolution of the Functional during Hospitalization

**Figure 1:**
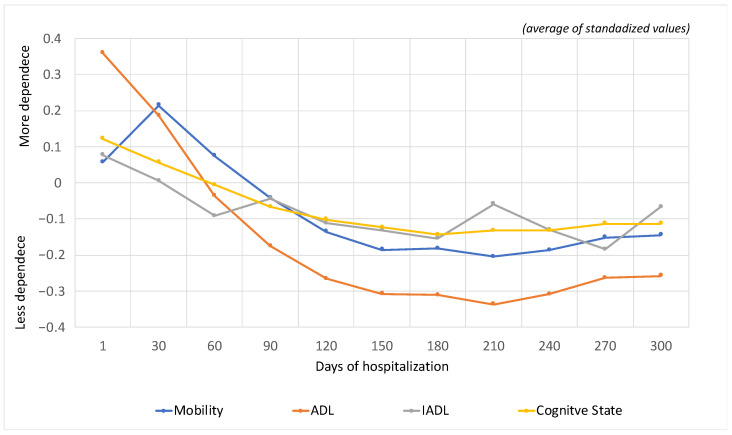
Synthetic indices: mobility, basic activities of daily living, instrumental activities of daily living and cognitive status in the Medium Term and Rehabilitation Units (mean of standardized values).

Figure 1 shows the evolution of the population’s functional profile throughout hospitalization, with assessments periodized every 30 days from the time of admission. Significant differences are observed in the dependence profile in mobility (F(119,324168) = 12.636; *p* < 0.001), basic activities of daily living (F(119,328779) = 19.937; *p* < 0.001), instrumental activities of daily living (F(95,71842) = 7.767; *p* < 0.001) and cognitive status (F(119,324163) = 10.369; *p* < 0.001).

### 3.3. Dependence Clusters

**Figure 2:**
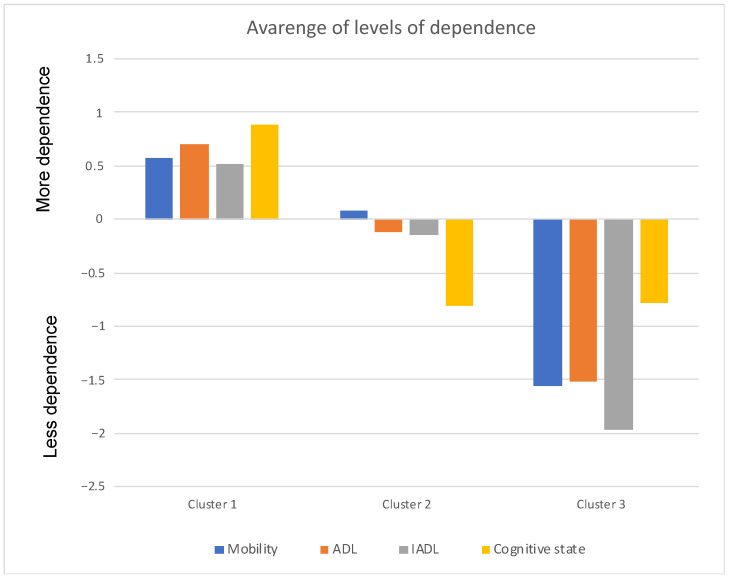
Mean of the dependence levels of Cluster 1, Cluster 2 and Cluster 3 of the Long Term Care and Maintenance Units.

In the Medium Term and Rehabilitation Units, the following partition was extracted:

(a) Cluster 1: Older adults with a higher degree of dependence (severe/complete self-care deficit);
(b) Cluster 2: Older adults with an intermediate degree of dependence (moderate self-care deficit);
(c) Cluster 3: Older adults with a lower degree of dependence (mild self-care deficit);

The three Clusters, on average, are quite visible following the configuration shown in Figure 2.

Cluster 1—47.1% (N = 25,321); Cluster 2—43.1% (N = 23,147); Cluster 3—9.8% (N = 5246). These differences are statistically significant in the dimensions related to mobility (F(2.53711) = 22,457.546; *p* < 0.001), ADL (F(2.53711) = 29,757.943; *p* < 0.001); IADL (F(2.53711) = 27,270.518; *p* < 0.001) and cognitive status (F(2.53711) = 77,341.434; *p* < 0.001).

**Figure 3:**
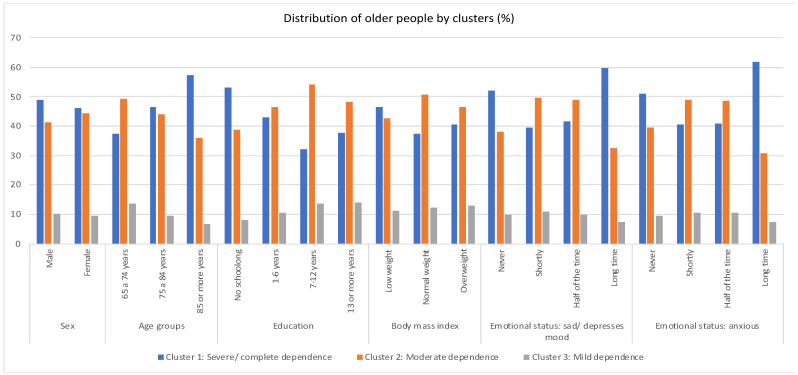
Distribution of the people aged 65 years or older in the Long Term Care and Maintenance Units by sex, age groups, educational level, body mass index, emotional state: sad/depressed and emotional state: anxious, per cluster.

Figure 3 shows the difference between the three clusters and the variables gender, age, education, body mass index and emotional state.

Table 2 shows the distribution of variables within each cluster.

1. Cluster 1 (severe dependence): a determinant of the need for totally compensatory nursing care is formed by a higher percentage of males aged 85 years or older. It is made up of people who have not attended school, are low-weight and feel depressed and anxious for a long time.
2. Cluster 2 (moderate dependence): where partially compensatory nursing care is required includes females aged between 65 and 84 years old, with schooling from 7 to 12 years old, normal body mass index and feeling of sadness or anxiety little or half of the time.
3. Cluster 3 (Mild dependence): who require nursing care at the psychoeducational level, predominantly male, aged 65 to 74, with 13 or more years of schooling. They are overweight, feel depressed a little of the time and feel anxious half of the time.

## 4. Discussion

In Medium-Duration Rehabilitation Units, within the first 90 days of hospitalization (maximum recommended length of stay), a decrease in dependence in mobility, activities of daily living and instrumental activities of daily living and an improvement in cognitive status are observed. Regarding the improvement in cognition, studies have already indicated improvements in cognition in older adults using aerobic dances [18]. The explanation for this mismatch between improved cognition and mobility lies in the fact that cognition itself is a dimension of mobility; in other words, after improving cognition, it is easier to achieve gains in mobility, like the body-mind connection [19]. However, older adults can remain hospitalized for more days, as long as clinically justified, with an improvement in all dimensions up to 210 days (maximum recommended time), since after this period, they decline. In ADL and mobility, the greatest amplitude of improvement was observed. IADL and cognitive status showed a slower and less pronounced improvement over time.

When these results are compared to those obtained in the study conducted in the NNICC Convalescence Units, we found that there was an improvement in all domains during the first 30 days of hospitalization [20]. It should be noted that in the convalescence units, people with less dependency and needing less rehabilitation time are referred, for example, for femur neck fracture. On the other hand, in the medium-duration units, people with situations that lead to a higher degree of initial dependence and need more rehabilitation time are referenced. So, in medium-duration units, the pattern of recovery is slower and more difficult to re-establish. This data is in line with the findings of some authors who show that interventions focused on the rehabilitation of hospitalized patients show improvements at the functional level, promoting independence after acute hospital admission [21,22].

In order to reinforce the findings obtained in this research, a study that analyzed the evolution of functioning in the NNICC convalescence units also showed improvements in the dimensions of functioning in the first 30 days of hospitalization. However, individuals in need of longer rehabilitation time would have to continue this program at home, although often, the supply of rehabilitation programs at home is scarce. The MTRUs, through the data presented in this article, thus show to be an option for hospitalization that promotes the rehabilitation of individuals when they need more than 30 days of hospitalization.

Makino et al. (2020) [23] warn that the limitation in performing instrumental activities is a strong indicator of moderate cognitive decline, which is described as a transitory phase between the typical changes of aging and the onset of dementia since they are activities that require memory and complex thinking [24]. Additionally, when the deterioration of the cognitive state is installed, its reversal is difficult and slow [25], which explains its recovery curve with a smaller amplitude in terms of functional improvement. Thus, it can be inferred that there is a close relationship between instrumental activities and the cognitive state.

Observing the age variable, the individuals who were older than 85 years were in the severe/complete dependence group, while those whose ages were between 65 and 84 years were in the moderate dependence group. Finally, most individuals between the ages of 65 and 74 are in the mild dependence group, thus confirming the studies that conclude that the older the individual, the higher the levels of dependence [20,26,27,28]. The same was found when analyzing the data from the NNICC Convalescence Units in Portugal [20].

When analyzing the clusters through the gender variable, it is concluded that males show a higher percentage in the “Severe/Complete” and “Mild” dependence clusters, while in the “Moderate” dependence cluster, females are more prevalent. This is an issue that still generates controversy because, although some studies corroborate these results and state that men are more dependent than women [29], others suggest the opposite [26,27,30]. However, the study conducted in convalescent units confirms the male gender in the “Severe/Complete” dependence cluster [20].

With regard to schooling, the results obtained suggest that people who did not attend school are more likely to be in the “Severe/Complete” dependence cluster, while older people with seven years of schooling or more are more likely to be in the “Moderate” and “Mild” dependence clusters. These results are confirmed by most of the literature, which suggests the schooling factor is one of the main predictors of high dependence levels in advanced age stages, i.e., the higher the education of the individual, the lower the dependence levels in advanced stages of life [31,32]. Another study conducted in Brazil goes in the same direction, confirming that illiterate people were more dependent for instrumental activities of daily living [33]. These results are again identical to the study conducted in the convalescence units of the NNICC [20].

Low weight was found to be more prevalent in the “Severe/Complete” dependence cluster. Studies show a close relationship between nutritional status, basic life activities and cognitive function, with low weight and malnutrition being more associated with the deterioration of mental functions [34,35].

The analysis of the emotional state suggests that, in the “severe/complete” dependence cluster, there is a predominance of older people who felt anxious and depressed for a long time, while in the moderate dependence cluster and in the light dependence cluster, there was a predominance of older people who felt sad or anxious half of the time. These results value mental status as a risk factor for the development of dependence in older adults, as observed in a scoping review developed in England, which sought to identify the care needs of non-institutionalized older adults, concluding that these individuals presented care needs at the mental health level [2]. Other studies confirm the findings of this research, suggesting that the addition of mental imbalances substantially increases the rate of dependence and difficulty in satisfaction with the activities of daily living of the older adult [36].

As limitations of the study, we highlight the high complexity of the architecture of the database, which brought added difficulties in the analysis of the extracted information over the period of hospitalization. It required a long period of time, approximately one year and requiring several updates. The correlation with the pathologies of the individuals and other variables (such as polypharmacy and pressure ulcers, among others), which were not available in the big data, could have brought new information to the study in terms of the mobility profile.

## 5. Conclusions

The rehabilitation care during hospitalization in the medium-term care units of the integrated long-term care network in Portugal contributes to a decrease in mobility dependence, ADL and IADL and an improvement in cognitive status. Observing the length of stay, we concluded that hospital stays between 30 and 90 days are more efficient in the rehabilitation of hospitalized individuals.

It is also concluded that in the group of severe or complete dependence, men, the group of older adults over 85 years old, and those who did not attend school and have low body index are in greater numbers.

This study highlights the importance of education, body mass index and mental health as facilitators of rehabilitation and recovery from acute health/illness situations, providing a contribution to clinical practice.

We suggest that future studies should assess the mental health of people undergoing rehabilitation, given the importance it may have for physical rehabilitation, since the lack of motivation for rehabilitation may have an influence on the results in terms of dependence.

## Figures and Tables

**Table 1:** Socio-demographic characterization of the study participants.

Socio-Demographic Variables	n (%)
Age (years) 65–74 75–84 ≥85	14,498 (24.6) 28,414 (48.1) 16,101 (27.3)
Sex Female Male	34,215 (58.0) 24,798 (42.0)
Marital status Married Widowed Single Divorced Union of Fact Unknown	24,665 (46.6) 19,510 (36.9) 6332 (12.0) 2166 (4.1) 167 (0.3) 90 (0.2)
Education (years) No education 1 to 6 7 to 12 ≥13	10,933 (34.1) 18,564 (57.8) 1346 (4.2) 1248 (3.9)
Professional level Unqualified Qualified Intermediate Specialist	22,568 (70.3) 7498 (23.4) 1443 (4.5) 571 (1.8)
Region of Portugal Alentejo Algarve Centro Lisboa e Vale do Tejo Norte	4668 (8.3) 2761 (4.9) 15,233 (27.2) 18,663 (31.6) 14,778 (26.3)

**Table 2:** Characterization of the people aged 65 years or older in the Long Term Care and Maintenance Units by sex, age groups, educational level, body mass index, emotional state: sad/depressed and emotional state: anxious, within each cluster.

		Levels of Dependence (%)		
		Cluster 1 Severe Dependence	Cluster 2 Moderate Dependence	Cluster 3 Mild Dependence
Sex	Male	41.4	38.1	40.9
	Female	58.6	61.9	59.1
	TOTAL	100.0	100.0	100.0
Age groups	65–74 years	19.4	27.9	33.8
	75–84 years	48.4	50.0	48.1
	85 or more years	32.3	22.1	18.1
	TOTAL	100.0	100.0	100.0
Educational level	No schooling	43.5	33.6	31.8
	1–6 years	51.6	58.7	59.0
	7–12 years	2.3	4.0	4.5
	13 or more years	2.7	3.6	4.7
	TOTAL	100.0	100.0	100.0
Body mass index	Low weight	41.7	34.7	34.7
	Normal weight	26.8	32.8	30.1
	Overweight	31.5	32.6	35.2
	TOTAL	100.0	100.0	100.0
Emotional state: sad/depressed	Never	43.9	35.2	39.4
	Shortly	28.3	38.8	36.8
	Half of the time	13.8	17.7	15.4
	Long time	14.0	8.3	8.3
	TOTAL	100.0	100.0	100.0
Emotional state: anxious	Never	46.7	39.4	41.7
	Shortly	29.6	38.9	36.7
	Half of the time	11.8	15.3	14.6
	Long time	11.9	6.4	6.9
	TOTAL	100.0	100.0	100.0

## Data Availability

Data are available from the authors upon reasonable request and with permission of the University of Évora.
